# Maternal RSV vaccination to protect infants in Brazil: a model-based cost-effectiveness analysis for incorporation into the National Immunisation Program

**DOI:** 10.1016/j.lana.2025.101356

**Published:** 2025-12-22

**Authors:** Sophia Aguiar Monteiro Borges, Erick Ohanesian Polli, Ana Carolina Nonato, Natacha Cerchiari, Stéphane Verguet, Ana Marli Christovam Sartori, Patrícia Coelho de Soárez

**Affiliations:** a Departamento de Medicina Preventiva, Faculdade de Medicina, Universidade de Sao Paulo, Sao Paulo, SP, Brazil; b Harvard T H Chan School of Public Health, Harvard University, Boston, MA, USA; c Departamento de Infectologia e Medicina Tropical, Faculdade de Medicina, Universidade de Sao Paulo, Sao Paulo, SP, Brazil

**Keywords:** Vaccine, Respiratory syncytial virus, Maternal immunization, Cost-effectiveness analysis

## Abstract

**Background:**

In Brazil, respiratory syncytial virus (RSV) is the primary cause of lower respiratory tract infections (LRTI) in children under two years of age. Maternal immunization with the bivalent RSV prefusion F protein vaccine (RSVpreF) has demonstrated high efficacy in protecting infants during their first 6 months of life against RSV-LRTI. We assessed the cost-effectiveness of implementing maternal RSV immunization in Brazil.

**Methods:**

We utilised a decision tree model, following a birth cohort during their first year of life. The model compared two strategies: maternal vaccination and no vaccination, from both healthcare system and societal perspectives. Secondary data from Brazilian Health Information Systems, administrative databases and international literature were used. The primary outcome was the incremental cost-effectiveness ratio (ICER) expressed as incremental cost per disability-adjusted life year (DALY) averted, in 2023 USD. We applied a cost-effectiveness threshold of 8000 USD (40,000 BRL) per DALY based on Brazilian guidelines.

**Findings:**

Introduction of RSV vaccination for pregnant women at 50% coverage would prevent around 37,000 RSV cases annually, including 9400 hospitalizations and 28,000 outpatient visits. The program would avert 80 deaths and 1660 DALYs, with an incremental cost of $49,200,000 USD from the healthcare system perspective (ICER $29,700 per DALY averted) and $48,800,000 USD from the societal perspective (ICER $29,200 per DALY averted). These ICERs exceed the acceptable cost-effectiveness Brazilian threshold. To be considered cost-effective, the vaccine dose price would need to be around $12. In sensitivity analyses, vaccine price and efficacy were the most influential parameters, as testing their uncertainty ranges resulted in the largest changes (i.e., the widest range) in the ICER. In the probabilistic sensitivity analysis, the probability that the maternal RSV immunization program is cost-effective at the Brazilian threshold of $8000 per DALY averted was 0% from the healthcare system perspective and 6% from the societal perspective.

**Interpretation:**

Our findings indicate maternal RSV immunization could substantially reduce disease burden but would require significant price reduction to meet Brazil's cost-effectiveness threshold.

**Funding:**

10.13039/501100001807São Paulo Research Foundation (FAPESP), Pan American Health Organization (PAHO) and Brazilian National Council for Scientific and Technological Development (CNPq).

**Research in context**

**Evidence before this study**

We conducted a literature review to evaluate the state of research on the cost-effectiveness of maternal immunization against respiratory syncytial virus (RSV) and the associated disease burden in Brazil. Our sources included PubMed, Google Scholar, government websites, administrative databases, epidemiological bulletins from the Brazilian Ministry of Health, and technical reports from governmental bodies. We focused on studies published between January 2000 and December 2025. Key references included clinical trials of the bivalent RSV prefusion F protein vaccine (RSVpreF), Brazil's Hospital Information System (SIH-SUS) data, a global meta-analysis of RSV-associated acute lower respiratory tract infections (LRTI) in children, and a systematic analysis of the RSV burden in Latin America. The RSVpreF vaccine has demonstrated high efficacy in preventing severe RSV-related LRTI in infants during their first six months. Currently, the only preventive option available for high-risk infants is palivizumab, which is costly and limited in scope. For new technologies to be adopted by the Sistema Único de Saúde (SUS), they must undergo cost-effectiveness analyses. Although the National Committee for Health Technology Incorporation (CONITEC) in Brazil has recommended the integration of the RSVpreF vaccine into the National Immunization Program, its nationwide implementation will be guided by strategies negotiated between the Ministry of Health and the manufacturer. Our study provides evidence to support planning and negotiations by the Ministry of Health.

**Added value of this study**

This study analyzes the health and economic impact of introducing the RSV maternal immunization program in Brazil. Assuming 50% maternal vaccination coverage, our model projects reductions of 15% in RSV cases, 21% in hospitalizations, and 25% in infant deaths. The incremental cost-effectiveness ratios (ICERs) were of $29,700 from the health system perspective and $29,200 from a societal perspective. For the program to be considered cost-effective based on the threshold of $8000 per DALY averted, the vaccine price must be around $12. These insights are important for informing policymakers and stakeholders regarding the potential benefits and costs associated with maternal RSV immunization in Brazil.

**Implications of all the available evidence**

RSV significantly impacts public health in Brazil, particularly among infants during their first months of life. The RSVPreF vaccine presents an important opportunity to reduce this disease burden by offering protection when infants are particularly vulnerable to severe illness. However, the successful introduction of the vaccine requires careful price negotiations, especially given Brazil's limited financial resources. The cost-effective vaccine dose price ($12) is about one-third of the proposed price of $39.1 (195 BRL) proposed by the manufacturer. This study offers valuable evidence for guiding price negotiations with manufacturers and will support decision-making by the Brazilian Ministry of Health. Ultimately, focusing on pricing and resource allocation will be essential for effectively integrating this vaccine into public health strategies.

## Introduction

Respiratory Syncytial Virus (RSV) is the leading cause of bronchiolitis and pneumonia in infants worldwide, with an estimated 101,000 deaths of children under five years of age in 2019.^1^ The highest burden occurs among young infants, with approximately two-thirds of RSV hospitalizations occurring in the first four months of life. In Brazil, RSV is the primary cause of lower respiratory tract infections (LRTI) in children under two years of age.^2^ A study analyzing hospitalization data on RSV-associated bronchiolitis, from 2000 to 2019, revealed 616,000 hospitalizations among infants, with 73% occurring in those under six months.^3^

RSV also poses an important economic burden, with the estimated global cost for managing inpatient and outpatient RSV-related LRTI of approximately €4.82 billion (2017), with 65% of these costs in low- and middle-income countries, where hospitalizations accounted for 55% of expenditure.^4^ Prematurity and chronic lung disease are known risk factors for severe RSV infection,^5^ but most infants hospitalized with RSV-LRTI are born at full term without known risk factors. Since 2011, Brazil's public healthcare system (*Sistema Único de Saúde, SUS)* has offered palivizumab, a monoclonal antibody with activity against RSV, for high-risk groups such as premature infants (≤28 weeks gestation) in their first RSV season and children under two years-old with chronic lung disease or heart disease.^6^ However, palivizumab limitations include high cost and monthly administration during the RSV season.

Two recent developments in RSV prevention include maternal vaccination (MV) with the bivalent RSV prefusion F protein vaccine (RSVpreF), which shows approximately 70% efficacy against severe RSV-LRTI in infants during their first six months of life,^7^ and nirsevimab, a long-acting monoclonal antibody that requires a single dose during RSV season.^8^ RSVpreF vaccine administered to pregnant women in their third trimester provides protection to newborns during their most vulnerable period, through antibody placental transfer.^9^^,^^10^

To be adopted by SUS, new technologies must undergo health technology assessment, including economic evaluation, to inform decision-making. In April 2025, the National Committee for Health Technology Incorporation (*Comissão Nacional de Incorporação de Tecnologias no Sistema Único de Saúde, CONITEC*) recommended the introduction of both RSVpreF vaccine and nirsevimab in the National Immunization Program.^11^ Brazil plans to implement both interventions as a combined strategy, with universal maternal vaccination and nirsevimab substituting palivizumab for high-risk infants. This study aims to provide a cost-effectiveness analysis (CEA) of maternal RSV immunization. Our research brings economic evidence to support implementation strategies planning and price negotiations.

## Methods

### Cost-effectiveness model

**Fig. 1:**
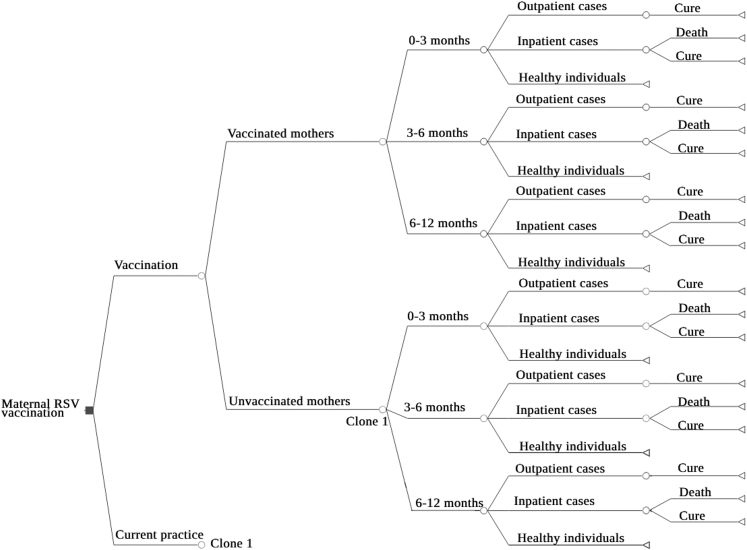
**Decision model structure for the cost-effectiveness evaluation of introducing universal maternal Respiratory Syncytial Virus (RSV) vaccination in Brazil.** RSV = Respiratory Syncytial Virus.

We utilized a decision tree model of a Brazilian birth cohort during their first year of life to assess RSV disease and economic burden and the cost-effectiveness of the RSV maternal vaccination (Fig. 1). This model drew from previous models,^12^^,^^13^ and compared two strategies: (i) year-round universal maternal vaccination with a single dose of RSVPreF vaccine administered from 28 to 36 6⁄7 weeks of gestational age; (ii) no vaccination.

We assumed the target population corresponds to all live births in Brazil in 2023. A total of 2,536,281 live births was used from the Brazilian Live Birth Information System (SINASC)^14^ as a proxy for pregnant women eligible for vaccination, representing an annual cohort. It was assumed that the current practice of palivizumab administration to premature infants during their first year of life, as well as children under two years-old with chronic lung disease or congenital heart disease, would remain unchanged under both strategies.

The model accounted only for the first RSV disease episode experienced by the child, considered the clinically most significant infection event. All RSV-attributable deaths were assumed to occur in hospitals as all severe cases were assumed to be hospitalized, and all RSV-LRTI cases were assumed to receive medical consultation within the Brazilian healthcare system.

We followed the birth cohort segmented into three age groups of: less than 3 months, 3 to less than 6 months, and 6 to less than 12 months. We evaluated cost-effectiveness from both the health system perspective, including direct medical costs covered by SUS, and the societal perspective, which included direct non-medical costs borne by patients (such as transportation) and indirect costs, including lost wages due to workdays off for caring for a child with RSV. Data were sourced from Brazil's National Health Information System databases, national administrative databases and peer-reviewed literature.

The analysis employed a one-year time horizon, reflecting that RSV maternal immunization primarily protects newborns during their initial months of life. We considered Brazil's 2023 life expectancy of 76.4 years^15^ and an annual discount rate of 5% for the years of life lost. All costs were converted from 2023 Brazilian Reais (BRL) to USD at the average annual exchange rate of 1 USD = 4.99 BRL in 2023.

The primary outcome was the incremental cost-effectiveness ratios (ICERs), incremental cost per disability-adjusted life year (DALY) averted, calculated as the difference in costs between the vaccination and no-vaccination scenarios divided by the difference in DALYs between these scenarios. We used disability weights for moderate LRTI of 0.051 for outpatient cases and disability weights for severe LRTI of 0.133 for inpatient cases, which were derived from the Global Burden of Disease (GBD) 2019 study,^16^ which estimated disability weights through population-based surveys using paired comparison methods across multiple countries. These weights quantify the degree of health loss associated with specific health outcomes and are used to calculate years lived with disability (YLD) in a population. They range from 0 (full health) to 1 (death).

Other outcomes of interest were hospitalizations averted, cases averted and deaths averted. Hospitalizations and outpatient cases averted were estimated by applying age-specific vaccine coverage and vaccine effectiveness to the Brazilian birth cohort and multiplying by the corresponding baseline rates of hospitalization and outpatient visits for each age group. Deaths averted were then calculated by applying age-specific case fatality rates to the number of hospitalizations averted in each group.

Brazil does not have a formally defined cost-effectiveness threshold per DALY for decision-making. Therefore, we used a threshold of 40,000 BRL ($8000) per QALY, as recommended by CONITEC.^17^

This study adhered to the 2022 Consolidated Health Economic Evaluation Reporting Standards (CHEERS) guidelines.^18^

Table 1 provides a list of the inputs incorporated into the model.

**Table 1:** Input parameters for the cost-effectiveness model of maternal RSV immunization in Brazil.

Parameter	Estimate	Source
Vaccine		
Vaccine coverage (%)	50%	Authors' assumption based on national vaccination coverage of pregnant women with DTPa and influenza in 2022 and 2023^14^^,^^19^
Vaccine efficacy (CI%)		
RSV-associated medically attended LRTI		
0–3 months	58% (31%–75%)	Authors estimates based on Simões et al., 2025,^7^ with exponential decay
3–<6 months	41% (31%–51%)	
≥6 months	0%	
Hospitalizations		
0–3 months	70% (37%–87%)	Authors estimates based on Simões et al., 2025,^7^ with exponential decay
3–<6 months	43% (26%–59%)	
≥6 months	0%	
Administration cost^a^	$2.6	Portnoy et al.^20^
Wastage rate (%)	5%	WHO^21^
Vaccine dose price^a^	39.1 USD/195 BRL	Latest dose price proposed by the manufacturer during negotiations with the Ministry of Health^22^
Epidemiology		
Annual birth cohort	2,536,281	SINASC
Birth distribution		
0–3 months	26%	SINASC
3–<6 months	26%	
6–<12 months	48%	
Life expectancy (years)	76.4	IBGE
RSV incidence rate (per 1000)		GB-ALRI-RSV study^1^
0–3 months	122	
3–<6 months	92	
6–<12 months	84	
RSV hospitalization rate (per 1000)		SIH-SUS, PNS, Bardach et al.^2^
<1 year	18	
0–3 months	28	
3–<6 months	21	
6–<12 months	11	
RSV length of inpatient stay (days)		SIH-SUS
0–3 months	6.6	
3–<6 months	6.1	
6–<12 months	5.6	
RSV in-hospital case-fatality rate (%)		SIH-SUS, SIM
0–3 months	0.9	
3–<6 months	0.7	
6–<12 months	0.4	
RSV outpatient rate (per 1000 live births)		Authors' estimates (total incidence–hospitalization rate)
0–3 months	94	
3–<6 months	71	
6–<12 months	73	
Disutility weights		
LRTI moderate cases	0.05 (0.03–0.07)	Global Burden of Disease Study 2021^16^
LRTI severe cases	0.13 (0.10–0.20)	Global Burden of Disease Study 2021^16^
Duration of illness (days)		Based on previous published studies 23
Inpatient case	10	
Outpatient case	5	
Direct medical costs^a^		
Outpatient costs per visit		Health resource utilization based on specialists' opinion, prices drawn from SIGTAP (adjusted by 2.7^24^) and BPS
Primary care (PC)	$9.6	
Emergency care (EC)	$17.3	
Final outpatient cost per case	$13.5	Authors assumption: 50% of outpatient cases in PC and 50% in EC
Inpatient costs		SIH-SUS (adjusted by 2.7^24^)
0–3 months	$585.3	
3–<6 months	$426.0	
6–<12 months	$318.8	
Laboratory tests costs		
Number of RT-PCR for RSV	56,254	GAL
Cost of RT-PCR	$63.8	SIGTAP (adjusted by 2.7^24^)
Direct non-medical costs^a^		
Transport costs per trip	$0.9	Own estimate based on average public transportation fares in Brazilian capital cities
Indirect costs^a^		
Average daily wage of women	$23.9	PNADc, IBGE
Working days	22	

BPS = Banco de Preços em Saúde (Health Price Database), SINASC = Sistema de Informação sobre Nascidos Vivos (Brazilian Live Birth Information System), IBGE = Instituto Brasileiro de Geografia e Estatística (Brazilian Institute of Geography and Statistics), SIH-SUS = Sistema de Informações Hospitalares do SUS (SUS Hospital Information System), SIM = Sistema de Informação sobre Mortalidade (Mortality Information System), GAL = Gerenciador de Ambiente Laboratorial (SUS Laboratory Management System), SIGTAP = Sistema de Gerenciamento da Tabela de Procedimentos, Medicamentos e Órteses/Próteses e Materiais Especiais–OPM do SUS (SUS Procedures, Medications and Orthoses/Prostheses and Special Materials Table Management System), PNADc = Pesquisa Nacional por Amostra de Domicílios Contínua (Continuous National Household Sample Survey).

a All costs were converted from 2023 Brazilian Reais (BRL) to United States Dollars (USD) at the average annual exchange rate of 1 USD = 4.99 BRL in 2023.

### Intervention

We evaluated an annual vaccination program targeting pregnant women in their third trimester (28–36 6/7 weeks) in alignment with recommendations from the WHO Strategic Advisory Group of Experts (SAGE) on Immunization.^25^ Vaccine coverage was estimated at 50%, based on current national tetanus diphtheria-acellular pertussis (Tdap) and influenza vaccination coverage among pregnant women, while also considering the restricted gestational period for administering the RSV vaccine. In 2022, the national average for Tdap coverage was 47%,^14^ whereas influenza vaccination rates for pregnant women varied between 50% and 60%, as reported in the 2023 vaccination campaign report.^19^

Vaccine efficacy (VE) inputs were derived from randomized controlled trial of RSVPreF.^7^ In our model we applied VE against RSV-associated hospitalization (70% at 90 days; 56% at 180 days) to inpatient cases and VE against RSV-associated medically attended LRTI (MA-LRTI) (57% at 90 days; 49% at 180 days) to outpatient cases. It was assumed that newborns are protected up to 6 months of age. We modeled waning immunity using an exponential decay function fitted to the available efficacy data points from the clinical trial. Detailed methodology for the VE modeling approach is provided in the Supplementary Material (Supplementary Table S1).

We did not include the consequences of vaccine adverse events, as reported rates were similar between vaccination and placebo groups and most events were mild to moderate.

### Disease burden estimates

We developed estimated rates of RSV outpatient visits, hospital admissions and deaths per 1000 per year for children aged less than 3 months, 3 to less than 6 months, and 6 to less than 12 months.

Our primary source of epidemiological data was the national hospitalization records for children under one year old, obtained from Brazil's Hospital Information System (*Sistema de Informações Hospitalares do SUS, SIH-SUS*) for 2023.^14^ To estimate the burden of RSV, we included all cases identified by RSV-specific ICD-10 codes (ICD: B97.4, J21.1, J20.5, J21.0) and 44% of lower respiratory tract infection (LRTI) hospitalizations identified by additional ICD-10 codes (ICD: J12, J12.8, J12.9, J21, J21.9). The latter proportion was derived from systematic review evidence regarding the attribution of RSV in LRTI cases in Brazil.^2^ Since SIH-SUS data only encompass hospitalizations in the public health sector, we scaled its counts by the share of children under one year who have private insurance (27%), as indicated by the National Health Survey (*Pesquisa Nacional em Saúde, PNS*).^26^ Public-sector hospitalizations were therefore divided by 0.73 (1–0.27) to estimate total admissions across both sectors. Rates were calculated per 1000 live births using data from the 2023 Brazilian Live Birth Information System (*Sistema de Informação sobre Nascidos Vivos, SINASC*).^14^

In the absence of a national data on RSV outpatient care or total incidence rates, we employed incidence rate estimates for upper-middle-income countries derived from a global meta-analysis of RSV-associated acute lower respiratory infections in children in 2019, referred in this paper as GB-ALRI-RSV study.^1^ The hospitalization rate for children under one year old in that study (18.7 per 1000 live births) aligned with our national estimate (18 per 1000 live births). We subsequently calculated the outpatient incidence rate for each age group by subtracting the hospitalization rate from the overall incidence rate for that age group. Our methodological choices were guided by a systematic review of studies related to RSV maternal vaccination. Other authors also used this approach in given the lack of national data.^23^

In-hospital case-fatality rate by age group was calculated by dividing the number of RSV deaths by the number of RSV hospitalizations, using 2023 data obtained from Brazil's Mortality Information System (*Sistema de Informação sobre Mortalidade, SIM*) and SIH-SUS.^14^ In the base case, we identified RSV deaths from death certificates where RSV-specific ICD codes or LRTI codes were listed as the underlying cause of death, attributing 44% of LRTI cases to RSV, consistent with the same attribution factor used for hospitalization estimates.

### Cost estimates

We obtained hospitalization costs from Brazil's SIH-SUS,^14^ which encompassed comprehensive expenses related to hospital services in the public sector, including personnel, medications, materials, and infrastructure. We calculated a weighted average of costs from RSV-specific ICD-coded cases and LRTI-coded cases, proportional to the number of cases registered in each category, for each age group. We assumed each hospitalized case involved an outpatient consultation at an emergency department, with a follow-up visit in 50% of the cases.

Outpatient costs were based on a package of health resource utilization developed through an expert panel consultation for primary care and emergency care scenarios. Unit costs were obtained from: the National Health Price Database (*Banco de Preços em Saúde, BPS*)^27^ and the Public Healthcare Payment System (*Sistema de Gerenciamento da Tabela de Procedimentos, Medicamentos e Órteses, Próteses e Materiais Especiais do SUS, SIGTAP*).^28^ We assumed an average of 1.5 medical visits per outpatient case to account for an initial visit for all cases and a follow-up visit in 50% of cases. Further details on the standardized care packages and their cost components are provided in the Supplementary Material (Supplementary Tables S2 and S3).

For laboratory costs, we incorporated 2023 RSV RT-PCR testing data from the National Laboratory Management System (*Sistema Gerenciador de Ambiente Laboratorial*, GAL), provided by the Brazilian Ministry of Health, which records all tests conducted in public health laboratories. We incorporated laboratory costs into inpatient expenses at a ratio of 1.2 tests per hospitalization, derived from the 56,000 RSV tests recorded in GAL and our estimated 45,400 hospitalizations. Due to the lack of specific RSV PCR costs in SIGTAP, we used the costs associated with HCV-PCR tests as a proxy.

All SIH-SUS and SIGTAP-based costs were multiplied by a factor of 2.7 to account for funding from federal, state, and municipal levels, as the original values reflected only federal funding. This adjustment aligns with the methodology from the Systems of Health Accounts (SHA) to identify the financial contributions from each SUS government level in healthcare services, based on data from 2015 to 2019.^24^

The vaccine cost included a dose price of $39.1 (based on the final price of 195 BRL proposed by the manufacturer during negotiations with the Ministry of Health, using an exchange rate of 4.99 BRL per USD), plus 5% of wastage rate and $2.6 of vaccine delivery costs.^20^^,^^21^^,^^29^ Direct non-medical costs included transportation expenses, calculated based on outpatient visits, hospitalization days, and average public transport fares across Brazilian capitals. The average public transport fare was estimated at $0.93 per trip, based on the average rates of public transportation in the state capitals of Brazil. For the societal perspective, wage loss was calculated using the average salary of women aged 15–49 years in households with children under one year old,^15^ with one day of lost wages ($23.9) assumed per healthcare visit or inpatient day.

### Sensitivity analyses

To evaluate the impact of parameter uncertainty, we performed univariate sensitivity analyses on key model inputs, particularly vaccine efficacy. We tested vaccine efficacy under several alternative assumptions. First, we replaced the base-case hospitalization VE with efficacy for severe medically attended lower respiratory tract infection, using 82% for 0–<3 months and 58% for 3–<6 months. We then applied lower and upper confidence intervals for both hospitalization VE (37–87% for 0–<3 months; 27–60% for 3–<6 months) and MA-LRTI VE (30–75% for 0–<3 months; 30–50% for 3–<6 months), based on cumulative VE for 0–6 months. We also tested an alternative waning pattern using linear decay instead of exponential decay (hospitalization: 70% for 0–<3 months and 40% for 3–<6 months; MA-LRTI: 58% for 0–<3 months and 40% for 3–<6 months). Additional analyses varied vaccine coverage (40% and 95%), vaccine dose price (75%, 50%, and 25% of the base value), and healthcare costs (increasing inpatient and outpatient costs by 25% and applying inpatient costs corresponding to the 90th percentile of reported hospitalization costs in the SIH-SUS database). We also varied hospitalization rates using the confidence interval for the proportion of LRTI cases attributable to RSV (44%, range 37–52%) and incorporated the confidence interval for national RSV incidence from the GB-ALRI-RSV study. Finally, for case-fatality rates, we expanded the definition to include deaths where RSV appeared in any field of the death certificate, not only as the underlying cause, to capture potential underreporting in death certificate completion practices.

We also conducted a probabilistic sensitivity analysis using Monte Carlo simulations with n = 1000 iterations, each selecting input parameter values from specified probability distributions. The distributions were selected based on both previously published RSV modeling studies and by analyzing the empirical distributions of our own data to determine the most appropriate fit for each parameter. Parameter values and distributions employed in sensitivity analyses are summarized in Supplementary Tables S4 and S5.

All analyses were conducted using R software (version 4.12.1). The code underlying this study are openly available and can be accessed via https://github.com/sophiaaguiar/vsrmodel.

### Ethical statement

The analysis utilized only anonymous secondary data, therefore, ethical approval was not required.

### Role of the funding source

The funders had no role in the study design, data collection, analysis, interpretation, manuscript preparation, or the decision to publish.

## Results

### Base case analysis

Table 2 presents the comparative clinical outcomes and economic impact of maternal RSV vaccination. Without vaccination, Brazil would experience around 243,000 annual RSV cases in infants, including 45,400 hospitalizations, 197,900 outpatient cases, and 317 deaths. Associated direct medical costs would reach $35,700,000 with indirect costs of $9,300,000. Total societal costs were higher in the vaccination scenario ($84,900,000) compared to the no vaccination scenario ($36,500,000) because they include the costs of implementing the vaccination program.

**Table 2:** Health outcomes and economic costs of maternal Respiratory Syncytial Virus (RSV) immunization versus no vaccination in Brazil.

	No vaccination	RSV maternal vaccination
**Population**		
Pregnant woman	2,536,281	2,536,281
Live births	2,536,281	2,536,281
**RSV health outcomes**		
RSV cases	243,300	206,400
RSV hospitalizations	45,360	36,000
RSV outpatient care	197,900	170,380
RSV deaths	317	238
Life years lost (discounted)	6498	4894
DALYs (discounted)	6800	5140
**Costs**		
Direct medical cost	$27,200,000	$21,000,000
Vaccination cost	–	$55,300,000
Indirect costs (wage loss)	$8,200,000	$7,600,000
Direct non-medical cost (transportation)	$1,200,000	$920,000
Societal total costs	$36,600,000	$85,000,000
Healthcare system total costs	$27,200,000	$76,300,000
**Differences (comparator = no vaccination)**		
RSV cases	–	−36,900
RSV hospitalizations	–	−9360
RSV outpatient care	–	−27,520
RSV deaths	–	−79
Life years lost (discounted)	–	−1604
DALYs (discounted)	–	−1660
Direct medical cost	–	−$6,100,000
Vaccination cost	–	$55,300,000
Indirect costs (transport and wage loss)	–	−$800,000
Societal total costs	–	$48,400,000
Healthcare system total costs	–	$49,200,000

RSV = Respiratory Syncytial Virus, DALY = Disability-adjusted life years.

RSV vaccination for pregnant women at 50% coverage would prevent 36,900 RSV cases annually, comprising 9360 fewer hospitalizations and 27,520 fewer outpatient visits. The program would avert 79 deaths and the loss of 1604 life years and 1660 DALYs. The estimated cost of the vaccination program would be $55,300,000, which is expected to generate savings of $6,100,000 in healthcare costs and $800,000 in indirect costs.

Table 3 shows the RSV maternal vaccination cost-effectiveness results. The ICER would be $29,700 per DALY averted from the healthcare system perspective and $29,200 per DALY averted from the societal perspective. To be considered cost-effective under the threshold of $8020 per DALY, the vaccine dose price would need to be $12 from the healthcare system perspective and $12.7 from the societal perspective.

**Table 3:** Cost-effectiveness results of maternal Respiratory Syncytial Virus (RSV) immunization in Brazil.

	Perspective	
	Healthcare system	Societal
Incremental cost	$49,200,000	$48,400,000
DALYs averted	1660	1660
Cost per DALY averted	$29,700	$29,200
Cost-effective vaccine dose price	$12	$12.7

DALY = Disability-adjusted life years.

### Sensitivity analysis

**Fig. 2:**
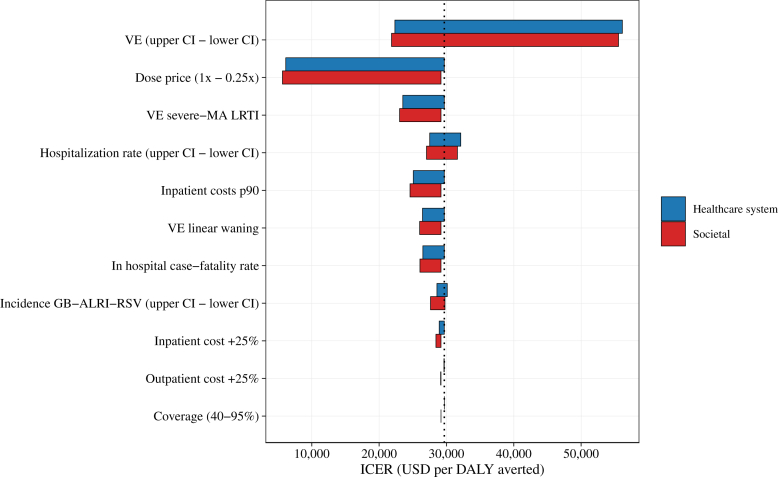
**Results of univariate sensitivity analysis of cost-effectiveness of Respiratory Syncytial Virus (RSV) maternal vaccination in Brazil (USD per DALY averted). (Blue)** healthcare system perspective; **(Red)** societal perspective. ICER = Incremental cost-effectiveness ratio, VE = Vaccine efficacy, MA-LRTI = Medically attended lower respiratory tract infection, p90 = 90th percentile, CI = confidence interval.

Tornado diagrams show the influence of parameter uncertainty on ICER results (Fig. 2). Vaccine dose price and vaccine efficacy were the most influential parameters. One-way sensitivity analysis tested the impact of vaccine efficacy by varying it across the bounds of its 95% Confidence Interval (CI). The lower 95% CI for vaccine efficacy resulted in an ICER of $56,100 per DALY averted (healthcare system perspective) and $55,700 per DALY averted (societal perspective), while the higher vaccine efficacy (upper 95% CI) reduced it to $22,300 and $21,800, respectively. These findings demonstrate that lower vaccine efficacy significantly reduces cost-effectiveness, while higher efficacy improves it.

Reducing the vaccine dose price by 75% substantially reduced the ICER to $6100 and $5,600, from healthcare system and societal perspectives, respectively, and a 50% reduction resulted in ICERs of $13,700 and $13,200. Using VE estimates for severe MA-LRTI, the ICER reduced to $23,500 and $23,000. Variations in hospitalization rates changed the ICER by approximately 8% in both directions, while higher case-fatality rates reduced the ICER value by approximately 11%, and different incidence estimates altered ICER base case results by only 1–5%.

**Fig. 3:**
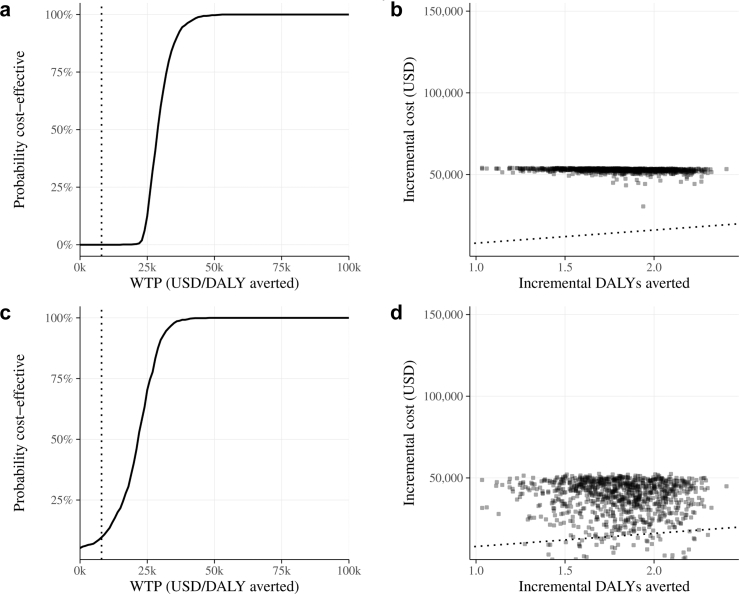
**Results of the probabilistic sensitivity analysis for the cost-effectiveness analysis of Respiratory Syncytial Virus (RSV) maternal vaccination in Brazil**. **(a)** Healthcare system perspective–cost-effectiveness acceptability curve; **(b)** Healthcare system perspective–cost-effectiveness plane; **(c)** Societal perspective–cost-effectiveness acceptability curve; **(d)** Societal perspective–cost-effectiveness plane. Note: dotted lines indicate the cost-effectiveness threshold of 8000 USD per DALY averted. DALYs = disability-adjusted life years, WTP = willingness-to-pay.

The probabilistic sensitivity analysis (n = 1000) showed that from the healthcare system perspective, the RSV maternal vaccination was not cost-effective in any interaction, as 0% fell below the threshold of $8000 per DALY averted. From the societal perspective, 6% of simulations fell below this threshold (Fig. 3). Results from the societal perspective show greater dispersion in the cost-effectiveness plane due to additional uncertainty in the length of stay and caregiver wages inputs.

## Discussion

This study evaluated the cost-effectiveness of introducing universal maternal immunization with RSV vaccine in Brazil. The results demonstrate that the RSV maternal vaccination program with 50% coverage would lead to reductions of approximately 15% in RSV cases, 21% in RSV hospitalizations, and 25% in RSV deaths among infants.

In our base case scenario, the vaccination program resulted in ICERs of $29,700 and $29,200 per DALY averted from the healthcare system and societal perspectives, respectively. To be considered cost-effective under the threshold of $8000 per DALY, the vaccine would have to be priced at around $12 (about 30% of the value proposed by the manufacturer). In the sensitivity analyses, the ICER ranged from $6100 to $56,100, from the healthcare system perspective, and from $5600 to $55,700, from the societal perspective.

Our findings have direct implications for vaccine introduction and price negotiations within Brazil's universal public healthcare system. The inclusion of the vaccine in the National Immunization Program was recently recommended by CONITEC, even though it was not considered cost-effective in a reassessment of the economic evaluation presented by the producer, conducted by the Health Technology Assessment Unit (*Núcleo de Avaliação de Tecnologias em Saúde, NATS*), which found an ICER of 29,670 USD (148,030 BRL) per QALY at the proposed dose price of 195 BRL.^22^

Notably, our sensitivity analyses demonstrate that vaccine price is the most influential ICER driver, with a 75% reduction in price significantly reducing the ICER to approximately one-fifth of the base case value. Conversely, using the lower CI value for VE significantly negatively impacted intervention cost-effectiveness, which underscores the importance of continuously assessing vaccine effectiveness post-implementation. These results align with findings from other studies of RSV maternal vaccination globally.^23^ In this systematic review, 17 of the 21 included studies indicated that maternal vaccination could be cost-effective depending on vaccine price and efficacy.^23^ Vaccination coverage levels did not affect ICER results, likely due to high implementation costs. However, achieving the Ministry of Health's 95% coverage target would reduce disease burden to approximately 173,200 RSV cases and 170 deaths annually, which corresponds to a 30% reduction in total RSV cases and a 47% reduction in RSV-related deaths, demonstrating a substantial public health impact that extends beyond cost-effectiveness considerations.

The comparison of ICERs from the societal perspective and the healthcare system perspective reveal minimal differences. This similarity may be attributed to the relatively short duration of work loss associated with RSV infections as it is an acute disease and Brazil's maternity leave policy (providing leave of up to four months).

In our base case, we employed VE for hospitalization outcomes (70% for 90-day cumulative efficacy) rather than the primary endpoint of severe MA-LRTI (82%). This decision was based on several considerations: the efficacy outcome for hospitalization reflects reductions in all RSV respiratory tract infections (i.e., not limited to LRTI) and aligns more closely with Brazil's hospitalization data, which includes both severe and non-severe cases. In Brazil, there are instances of hospitalization based on social criteria, for which decisions may not be made solely on clinical grounds. For instance, patients living in remote areas may be admitted because they may face difficulties returning to the healthcare facility for follow-up treatment due to transportation challenges.^30^^,^^31^ Furthermore, the severe MA-LRTI endpoint uses intensive care units (ICU) admission-like criteria that may not fully represent Brazil's hospital practices. In scenario analysis, using VE estimates for severe MA-LRTI yielded more favorable ICERs of around $23,000 per DALY averted, though still above the cost-effectiveness threshold at the current dose price.

Our study faced methodological challenges in estimating RSV disease burden. National RSV incidence was derived from 2019 upper-middle-income country estimates due to absence of Brazilian national data on outpatient care by specific diagnoses and limited routine RSV testing. Sensitivity analysis further exploring this uncertainty did not show substantial effects on ICER results. Additionally, while we accounted for private healthcare utilization based on insurance coverage among infants, we could not incorporate actual private sector treatment costs (due to unavailability), which may be substantially higher than public sector costs.^32^ Similarly, our laboratory testing data was limited to tests performed in the public sector, with no information available about testing volume in private facilities. These limitations highlight the need to strengthen Brazil's surveillance and health information systems, expand routine RSV testing, and implement systematic viral monitoring.

We did not include long-term consequences of RSV disease, such as recurrent wheezing and asthma, in our model, as the potential impact of preventive interventions like MV and monoclonal antibodies (mAb) on these long-term outcomes remains uncertain.^33^^,^^34^ In our model, we utilized the population of live births as a proxy for our target population of pregnant women and the birth cohort. However, this population does not align with our ideal target because vaccination occurs between 28 and 36 6/7 weeks of gestation. As a result, mothers of extremely preterm infants would not have received the vaccine, which would instead be reflected in the non-covered population in our model.

In societal perspective analyses, we did not account for out-of-pocket (OOP) expenses incurred by families for treatment, nor did we consider other costs related to child care, such as food and other direct non-medical expenses. Our estimates likely underestimate transportation costs, as they were based solely on public transport fares and did not account for private travel expenses. Another important limitation of our analysis is the lack of equity considerations, which is crucial given Brazil's economic, social, and regional disparities. Methodologies like extended cost-effectiveness analysis^35^ and distributional cost-effectiveness analysis^36^ incorporate equity considerations and financial risk protection into cost-effectiveness assessments. Future research should aim to incorporate these factors to provide a more holistic view of the economic burden of RSV.

Future research could also explore alternative model structures, such as Markov or state-transition models. These models may allow for a more explicit representation of disease progression, capturing repeated events (such as reinfections), age-and season-dependent variations in risk, recurrent RSV infections and associated healthcare use, as well as potential long-term outcomes (e.g., recurrent wheezing or asthma-like symptoms), which were not included in our static decision-tree framework. Incorporating these structures could improve estimation of lifetime costs and health effects and provide a more comprehensive assessment of the value of maternal RSV immunization.

RSV represents a significant health burden in Brazil, with particularly severe impacts on infants in their first months of life. Until recently, palivizumab was the only preventive measure available, restricted to high-risk infants in the public sector or accessible in private clinics at substantial costs (600–2200 USD). The adoption of maternal RSV immunization into the national public program addresses this critical window of vulnerability when infants are most susceptible to severe disease, offering protection during a period when active immunization is not yet feasible. However, the integration of high-cost medications into a public healthcare system requires careful consideration, given the limited financial resources and possible challenges with implementation.

In conclusion, our study emphasizes the potential of universal maternal immunization with the RSV vaccine to significantly mitigate the disease burden among infants in Brazil. However, our analysis highlights the critical importance of price negotiations, with sensitivity analyses indicating that the intervention would only be cost-effective at significantly lower prices, reinforcing the necessity for favorable pricing agreements that align with Brazil's resource constraints. The long-term program sustainability will depend on securing favorable pricing agreements that align with SUS resource constraints while ensuring equitable access to this important preventive intervention.

## Contributors

S.A.M.B., E.O.P., A.C.N., N.R.M.C., A.M.C.S. and P.C.S. conceived the study, designed the analytical plan, collected the data and interpreted the findings. These six authors also accessed and verified all underlying data. S.A.M.B., E.O.P. and A.C.N. performed the modelling analyses, with methodological advice from A.M.C.S., P.C.S. and S.V. S.A.M.B. drafted the first version of the manuscript, and A.M.C.S., P.C.S. and S.V. revised and edited subsequent drafts. A.M.C.S. and P.C.S. supervised the study. All authors approved the final manuscript and the decision to submit it for publication.

## Data sharing statement

This study relied on anonymised secondary data. Datasets are publicly available from their original sources. Laboratory-testing records were obtained from the National Laboratory Management System (Sistema Gerenciador de Ambiente Laboratorial—GAL), provided by the Brazilian Ministry of Health. The R code used in the analyses is openly available at https://github.com/sophiaaguiar/vsrmodel.

## Declaration of generative AI and AI-assisted technologies in the writing process

During manuscript preparation the authors used Anthropic Claude 4 Sonnet (accessed through Harvard's Sandbox AI, which provides a secure environment to explore Generative AI) solely to improve wording and readability of sentences already written by the authors. Example prompt: “Please shorten the following sentence for clarity while keeping the technical meaning: …” No text, figures, tables, or references were generated de novo by the tool. All output was reviewed and edited by the authors as needed, and the authors take full responsibility for the content of the publication.

## Declaration of interests

The authors declare no competing interests.

## Appendix A Supplementary data

Supplementary Figure and Tables

Translated Summary
