# Supplementary material for: Maternal RSV vaccination to protect infants in Brazil: a model-based cost-effectiveness analysis for incorporation into the National Immunisation Program
Source: Lancet Reg Health Am. 2025 Dec 22;53:101356. doi: 10.1016/j.lana.2025.101356 (PMC12800478; doi:10.1016/j.lana.2025.101356)
Supplement: Supplementary Figure and Tables [file mmc1.pdf]

Supplementary Material - Maternal RSV Vaccination to Protect Infants in Brazil: Health Impact, Cost-Effectiveness, and Price-Negotiation Implications for the National Immunization Program

Table of contents

1. Vaccine efficacy waning 2

Supplementary Table S1. Vaccine efficacy used in the model of cost-effectiveness evaluation of pregnant women immunization with rsv vaccine in brazil, by age group and outcome 3

Supplementary Figure S1. Exponential decay models of RSVpreF vaccine efficacy over time for different clinical outcomes 4

2. Outpatient costs 5

Supplementary Table S2. Primary Care Outpatient Package for RSV Case Management 6

Supplementary Table S3. Emergency Care Outpatient Package for RSV Case Management 7

3. Sensitivity Analyses 8

Supplementary Table S4. Parameters and ranges used in univariate sensitivity analyses 8

Supplementary Table S5. Parameter Values and Distributions for Probabilistic Sensitivity Analysis 9

4. References 10

1. Vaccine efficacy waning

Vaccine efficacy (VE) for hospitalization and medically-attended lower tract respiratory infection (MA-LRTI) outcomes were used in the base case model, because they fit better to our hospitalization data; and VE for severe MA-LRTI (instead of VE for hospitalization) was used in the sensitivity analyses. In the base case model, VE waning was modelled using an exponential function fitted to cumulative efficacy data from the clinical trial,[1] as illustrated in Figures S1-S3. The formula $VE(t) = a\cdot e^{(-b\cdot t)}$ was employed, where VE(t) represents VE at time t (months), with parameters a and b estimated from trial data. Linear decay models were tried in sensitivity analyses (Table S1).

For the time periods reported in the clinical trial, we assigned the cumulative efficacy values to their corresponding midpoints (e.g., 90-day cumulative efficacy was assigned to 1.5 months, 120-day to 2.0 months, etc.), shown as observed data points in Figures S1-S3. To calculate average efficacy for the critical 3-6 month period, we used the integral of the exponential function over this time interval:

$$VE\_avg(3\text{-}6) = (1/3)\cdot\int_3^6 a\cdot e^{(-b\cdot t)}\,dt = (a/3b)\cdot(e^{(-3b)} - e^{(-6b)}) \qquad (1)$$

As shown in Table S1, VE for 0-<3 months represents observed clinical trial data. Efficacy for 3-<6 months was calculated as average efficacy using an exponential decay model fitted to the trial data, with horizontal dotted lines in Figures S1-S3 representing these average efficacies and their confidence intervals. No protection was assumed after 6 months of life.

Supplementary Table S1. Vaccine Efficacy used in the model of Cost-effectiveness evaluation of pregnant women immunization with RSV vaccine in Brazil, by Age Group and Outcome

Outcome	Age group	Exponential decay model VE (95% CI)	Linear decay model VE (95% CI)
Hospitalization	0-3 months	70% (37%-87%)	70% (37%-87%)
	3-<6 months	43% (27%-60%)	40% (30%-50%)
	≥ 6 months	0%	0%
MA LRTI	0-3 months	58% (30%-75%)	58% (30%-75%)
	3-<6 months	40% (30%-50%)	40% (30%-50%)
	≥ 6 months	0%	0%
Severe MA-LRTI	0-3 months	82% (58%-94%)	82% (58%-94%)
	3-<6 months	58% (32%-84%)	-
	≥ 6 months	0%	0%

CI = confidence interval, LRTI = lower respiratory tract infection, MA = medically attended, RSV = respiratory syncytial vírus, VE = vaccine efficacy

Supplementary Figure S1. Exponential decay models of RSVpreF vaccine efficacy over time for different clinical outcomes: (a) RSV-associated hospitalization, (b) medically attended RSV lower respiratory tract infection (RSV MA-LRTI), and (c) severe medically attended RSV lower respiratory tract infection (severe RSV MA-LRTI).

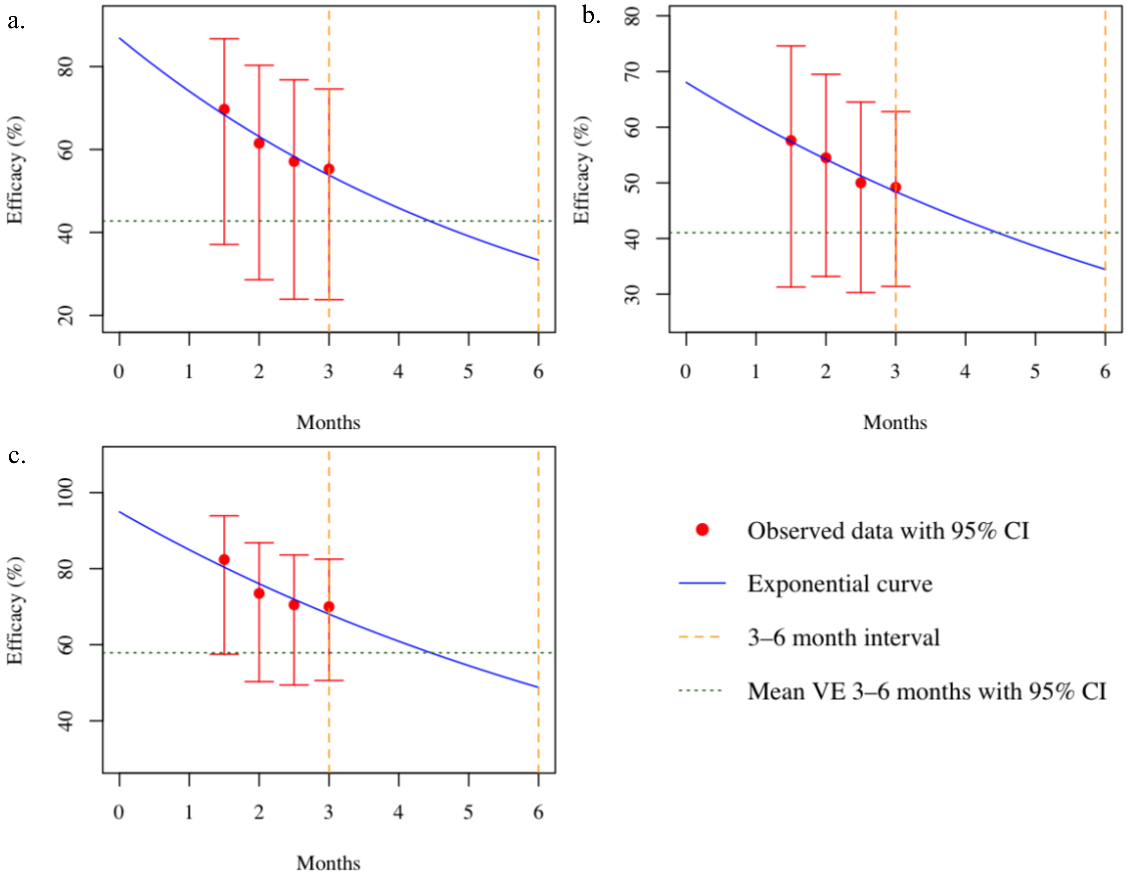

a. RSV-associated hospitalization, b. medically attended RSV lower respiratory tract infection (RSV MA-LRTI), and c. severe medically attended RSV lower respiratory tract infection (severe RSV MA-LRTI)

VE = vaccine efficacy, MA-LRTI = medically attended lower respiratory tract infection, CI = confidence interval

2. Outpatient costs

In the absence of comprehensive Brazilian data on outpatient care according to diagnoses, expert consultation was used to develop a standardized outpatient care packages to estimate outpatient costs. Information was gathered via an online survey distributed to pediatricians, general practitioners, pulmonologists, infectious disease specialists, and family doctors providing healthcare at SUS services across Brazil, through professional networks, social media, and messaging apps. The survey included questions about diagnostic tests, prescriptions, and treatments commonly used for RSV-related cases at different healthcare levels.

Based on responses from 24 participating physicians, we constructed two outpatient care packages: one for primary care settings (Table S2) and another for emergency care units (Table S3). Unit costs for each component were obtained from the National Health Price Database (BPS - Banco de Preços em Saúde)[2] and the Public Healthcare Payment System (SIGTAP - Sistema de Gerenciamento da Tabela de Procedimentos do SUS).[3] For primary care consultations, we applied the same unit cost as emergency care visits due to the absence of specific primary care pricing in SIGTAP. All SIGTAP-based costs were multiplied by a factor of 2.66 to account for funding from federal, state, and municipal levels.[4]

The inclusion of bronchodilators in the emergency care package reflects their common use in clinical practice, although the updated Brazilian Society of Pediatrics guidelines[5] discourage their routine use in RSV cases.

Supplementary Table S2. Primary Care Outpatient Package for RSV Case Management

**Primary care**			
**Outpatient item**	**SIGTAP/BPS Product**	**Quantity**	**Unit price (USD / BRL)**
Medical consultation	Medical consultation in primary care	1.5	6 / 29
In-facility prescription			
Nasal saline solution	Sodium chloride 0.9% (30 or 10ml)	1.0	0.2 / 1
Antipyretic	Dipyrone - drops 500mg/ml (10ml bottle)	1.0	0.2 / 1
Home prescription			
Nasal saline solution	Sodium chloride 0.9% (30 or 10ml)	1.0	0.2 / 1
Antipyretic	Dipyrone - drops 500mg/ml (10ml bottle)	1.0	0.2 / 1
Total (USD)			9.6

BPS = Banco de Preços em Saúde (Health Price Database), SIGTAP = Sistema de Gerenciamento da Tabela de Procedimentos, Medicamentos e OPM do SUS (Management System for SUS Procedures, Medications and Medical Materials Table).

Supplementary Table S3. Emergency Care Outpatient Package for RSV Case Management

Emergency care<br>Outpatient item	SIGTAP/BPS Product	Quantity	Unit price (USD / BRL)	Source
Medical consultation	Medical care in Emergency Care Unit	1.5	6 / 29	SIGTAP
Chest X-ray	Chest X-ray (PA)	1	4 / 18	SIGTAP
Complete blood count	Complete blood count	1	2 / 11	SIGTAP
In-facility prescription				
Nasal saline solution	Sodium chloride 0.9% (30 or 10ml)	1	0.2 / 1	BPS
Bronchodilator (BPS)	Salbutamol 100mcg/dose (oral aerosol) - 200 doses bottle	1	2 / 9	BPS
Nebulization with hypertonic solution	Inhalation with hypertonic solution - 3% (30 or 25ml)	1	0.3 / 1.3	BPS
Home prescription				
Antipyretic	Dipyrone - drops 500mg/ml (10ml bottle)	1	0.2 / 1	BPS
Nasal irrigation	Sodium chloride 0.9% (30 or 10ml)	1	0.2 / 1	BPS
Total (USD)			17.3	

BPS = Banco de Preços em Saúde (Health Price Database), SIGTAP = Sistema de Gerenciamento da Tabela de Procedimentos, Medicamentos e OPM do SUS (Management System for SUS Procedures, Medications and Medical Materials Table).

3. Sensitivity Analyses

Supplementary Table S4. Parameters and ranges used in univariate sensitivity analyses

Input	Base Case	Variation description and values	Source
Vaccine efficacy	Values in Table S1	Linear waning; severe medically attended LRTI; Lower and upper bounds from 95% CI (Values in Table S1)	Simoes et al.[1]
Vaccine coverage	50%	40%, 95%	Authors assumption and Ministry of Health coverage target
Vaccine dose price	$39.1	75%,50% and 25% of the price	
Inpatient cost		Inpatient costs for the 90th percentile	SIH-SUS
0-3 months	$585.3	$1625	
3-<6 months	$426.0	$760	
≥ 6 months	$318.8	$403	
Inpatient cost		Increased inpatient costs by 25%	Authors assumption
0-3 months	$585.3	$732	
3-<6 months	$426.0	$533	
≥ 6 months	$318.8	$400	
Outpatient costs per visit (SUS)		Increased outpatient costs by 25%	Authors assumption
Primary care (PC)	$9.6	$12	
Emergency care (EC)	$17.3	$21.6	
Final outpatient cost per case	$13.5	$16.9	
Proportion of LRTI attributable to RSV	44%	Lower 36.5%, Upper 52% (95% CI)	Systematic Review on RSV disease burden in Latin America[6]
RSV-LRTI national incidence		Lower and upper bounds from 95% CI	GB-ALRI-RSV study[7]
0-3 months	121.5	(56 – 264)	
3-<6 months	91.6	(29 – 292)	
≥ 6 months	84.3	(40 – 180)	
RSV in-hospital case-fatality rate (%)		Include RSV mentions in any field of the death certificate	SIH-SUS, SIM
0-3 months	0.9	1	
3-<6 months	0.7	0.8	
≥ 6 months	0.4	0.5	

RSV = respiratory syncytial virus, LRTI = lower respiratory tract infection, MA-LRTI = medically attended lower respiratory tract infection, CI = confidence interval, SUS = Sistema Único de Saúde (Brazilian Universal Health System), SIH-SUS = Sistema de Informações Hospitalares do SUS (Hospital Information System of SUS), SIM = Sistema de Informação sobre Mortalidade (Mortality Information System), PC = primary care, EC = emergency care.

Suppelementary Table S5. Parameter Values and Distributions for Probabilistic Sensitivity Analysis

Input	Distribution	Parameters
Inpatient cost for RSV coded cases		
0-3 months	Log-normal	mean (log)= 4.47; SD (log)= 1.17
3-<6 months	Log-normal	mean (log)= 4.27; SD (log)= 1.00
≥ 6 months	Log-normal	mean (log)= 4.17; SD (log)= 0.85
Inpatient cost for LRTI coded cases		
0-3 months	Log-normal	mean (log)= 4.36; SD (log)= 1.06
3-<6 months	Log-normal	mean (log)= 4.21; SD (log)= 0.91
≥ 6 months	Log-normal	mean (log)= 4.17; SD (log)= 0.80
Length of stay for RSV coded cases		
0-3 months	Log-normal	mean (log)= 1.72; SD (log)= 0.63
3-<6 months	Log-normal	mean (log)= 1.64; SD (log)= 0.59
≥ 6 months	Log-normal	mean (log)= 1.58; SD (log)= 0.55
Length of stay for LRTI coded cases		
0-3 months	Log-normal	mean (log)= 1.62; SD (log)= 0.64
3-<6 months	Log-normal	mean (log)= 1.56; SD (log)= 0.60
≥ 6 months	Log-normal	mean (log)= 1.50; SD (log)= 0.56
Disutilities		
DALY moderate	Beta	α = 23.66; β = 440.17
DALY severe	Beta	α = 25.16; β = 152.47
Vaccine Efficacy for Hospitalization		
0-3 months	Beta	α = 7.82; β = 3.93
3-<6 months	Beta	α = 14.44; β = 19.55
≥ 6 months	Fixed	0
Vaccine Efficacy for Outpatient		
0-3 months	Beta	α = 10.24; β = 8.40
3-<6 months	Beta	α = 40.37; β = 58.26
≥ 6 months	Fixed	0
Incidence RSV-GB-LRTI-2019		
0-3 months	Beta	α = 4.48; β = 32.36
3-<6 months	Beta	α = 1.60; β = 15.91
≥ 6 months	Beta	α = 4.97; β = 54.03
Caregiver wages	Log-normal	mean (log)= 5.85; SD (log)= 0.90

RSV = Respiratory Syncytial Virus, LRTI = lower respiratory tract infection, SD = standard deviation, DALY = disability-adjusted life year.

References

1. Simões EAF, Pahud BA, Madhi SA, Kampmann B, Shittu E, Radley D, et al. Efficacy, safety, and immunogenicity of the MATISSE (Maternal Immunization Study for Safety and Efficacy) maternal respiratory syncytial virus prefusion F protein vaccine trial. Obstet Gynecol. 2025;145(2):157.
2. BPS - Banco de Preços em Saúde [Internet]. [cited 2025 Mar 21]. Available from: https://bps-legado.saude.gov.br/login.jsf
3. SIGTAP - Sistema de Gerenciamento da Tabela de Procedimentos, Medicamentos e OPM do SUS [Internet]. [cited 2025 Mar 21]. Available from: http://sigtap.datasus.gov.br/tabela-unificada/app/sec/inicio.jsp
4. Ministério da Saúde, Fundação Oswaldo Cruz. Contas de saúde na perspectiva da contabilidade internacional: Conta SHA para o Brasil, 2015 a 2019. Rio de Janeiro: Ministério da Saúde; 2021.
5. Sociedade Brasileira de Pediatria. Diretrizes para o manejo da infecção causada pelo vírus sincicial respiratório (VSR) - 2017 [Internet]. Departamentos Científicos de Cardiologia, Imunizações, Infectologia, Neonatologia e Pneumologia; 2017 [cited 2025 May 11]. Available from: https://www.sbp.com.br/fileadmin/user_upload/Diretrizes_manejo_infeccao_causada_VSR2017.pdf
6. Bardach A, Rey-Ares L, Cafferata ML, Cormick G, Romano M, Ruvinsky S, et al. Systematic review and meta-analysis of respiratory syncytial virus infection epidemiology in Latin America. Rev Med Virol. 2014;24(2):76-89.
7. Li Y, Wang X, Blau DM, Caballero MT, Feikin DR, Gill CJ, et al. Global, regional, and national disease burden estimates of acute lower respiratory infections due to respiratory syncytial virus in children younger than 5 years in 2019: a systematic analysis. Lancet. 2022;399(10340):2047-64.
