# Supplementary material for: Maternal RSV vaccination to protect infants in Brazil: a model-based cost-effectiveness analysis for incorporation into the National Immunisation Program
Source: Lancet Reg Health Am. 2025 Dec 22;53:101356. doi: 10.1016/j.lana.2025.101356 (PMC12800478; doi:10.1016/j.lana.2025.101356)
Supplement: Translated Summary [file mmc2.pdf]

Editorial disclaimer: *This translation in Portuguese was submitted by the authors and we reproduce it as supplied. It has not been peer reviewed. Our editorial processes have only been applied to the original abstract in English, which should serve as reference for this manuscript.*

TRANSLATED SUMMARY

Contexto: No Brasil, o vírus sincicial respiratório (VSR) é a principal causa de infecções do trato respiratório inferior (ITRI) em crianças menores de dois anos. A imunização materna com a vacina bivalente de proteína F pré-fusão do VSR (RSVpreF) demonstrou alta eficácia em proteger lactentes nos primeiros 6 meses de vida contra VSR-ITRI. Avaliamos a custo-efetividade da imunização materna contra o VSR no Brasil.

Métodos: Utilizamos um modelo de árvore de decisão, acompanhando uma coorte de nascidos vivos durante o primeiro ano de vida. O modelo comparou duas estratégias: vacinação materna e não vacinação, sob as perspectivas do sistema de saúde e da sociedade. Foram utilizados dados secundários dos Sistemas de Informação em Saúde, bases administrativas brasileiras e literatura internacional. O desfecho primário foi a razão incremental de custo-efetividade (RCEI), expressa como custo incremental por ano de vida ajustado por incapacidade (DALY) evitado, em dólares de 2023. Aplicamos um limiar de custo-efetividade de 8.000 USD (40.000 BRL) por DALY, baseado nas diretrizes brasileiras.

Resultados: A introdução da vacinação contra VSR para gestantes, com 50% de cobertura, evitaria cerca de 37.000 casos anuais de VSR, incluindo 9.400 hospitalizações e 28.000 atendimentos ambulatoriais. O programa evitaria 80 óbitos e 1.660 DALYs, com custo incremental de 49.200.000 USD sob a perspectiva do sistema de saúde (RCEI de 29.700 USD por DALY evitado) e 48.800.000 USD sob a perspectiva societal (RCEI de 29.200 USD por DALY evitado). Essas RCEIs ultrapassam o limiar brasileiro de custo-efetividade. Para ser considerada custo-efetiva, a vacina precisaria ter preço de aproximadamente 12 USD por dose. Nas análises de sensibilidade, o preço e a eficácia da vacina foram os parâmetros mais influentes, apresentando as maiores variações na RCEI. Na análise de sensibilidade probabilística, a probabilidade de que o programa de imunização materna seja custo-efetivo ao limiar brasileiro de 8.000 USD por DALY evitado foi de 0% na perspectiva do sistema de saúde e 6% na perspectiva societal.

Interpretação: Os achados indicam que a imunização materna contra o VSR pode reduzir substancialmente a carga da doença, mas exigiria uma redução significativa de preço para atender ao limiar de custo-efetividade no Brasil.

Financiamento: S.A.M.B. recebeu apoio da Fundação de Amparo à Pesquisa do Estado de São Paulo (FAPESP; processo nº 2024/07223-0). E.O.P., A.C.N., N.R.M.C., A.M.C.S. e P.C.S. receberam suporte por meio de um acordo com a Organização Pan-Americana da Saúde (OPAS) (“Carta-Acordo OPAS”, nº SCON2024-00174).
